# Contralesional functional network reorganization of the insular cortex in diffuse low-grade glioma patients

**DOI:** 10.1038/s41598-020-79845-3

**Published:** 2021-01-12

**Authors:** Shengyu Fang, Chunyao Zhou, Yinyan Wang, Tao Jiang

**Affiliations:** 1 Beijing Neurosurgical Institute, Capital Medical University, Beijing, China; 2 Department of Neurosurgery, Beijing Tiantan Hospital, Capital Medical University, 119, the Western Road of the southern 4th Ring Road, Beijing, 100070 China; 3 Research Unit of Accurate Diagnosis, Treatment, and Translational Medicine of Brain Tumors Chinese (2019RU11), Chinese Academy of Medical Sciences, Beijing, China

**Keywords:** Cognitive neuroscience, Diseases of the nervous system, Neural circuits, Cancer imaging

## Abstract

Diffuse low-grade gliomas (DLGGs) growing on the insular lobe induce contralesional hemispheric insular lobe compensation of damaged functioning by increasing cortical volumes. However, it remains unclear how functional networks are altered in patients with insular lobe DLGGs during functional compensation. Thirty-five patients with insular DLGGs were classified into the left (insL, n = 16) and right groups (insR, n = 19), and 33 healthy subjects were included in the control group. Resting state functional magnetic resonance imaging was used to generate functional connectivity (FC), and network topological properties were evaluated using graph theoretical analysis based on FC matrices. Network-based statistics were applied to compare differences in the FC matrices. A false discovery rate was applied to correct the topological properties. There was no difference in the FC of edges between the control and insL groups; however, the nodal shortest path length of the right insular lobe was significantly increased in the insL group compared to the control group. Additionally, FC was increased in the functional edges originating from the left insular lobe in the insR group compared to the control group. Moreover, there were no differences in topological properties between the insR and control groups. The contralesional insular lobe is crucial for network alterations. The detailed patterns of network alterations were different depending on the affected hemisphere. The observed network alterations might be associated with functional network reorganization and functional compensation.

## Introduction

Gliomas frequently infiltrate eloquent areas and subcortical fibers thereby inducing neurological deficits. However, the invasion of a glioma does not always induce detectable neurological deficits due to the functional plasticity of the brain^1^. It is generally considered that normal cortices surrounding the lesion are first recruited for functional reorganization to compensate for the impaired neurological functions^2^. Moreover, if the surrounding cortices show insufficient functional plasticity, the contralateral cortex in the symmetric area of the lesion will be recruited^3,4^. However, some previous studies have suggested that network reorganization and morphological remodeling does not necessarily lead to functional compensation, and might be intrinsic alterations following the appearance of lesions involving the eloquent area^5^. Importantly, accumulating studies have reported that functional plasticity not only depends on morphological remodeling^6,7^ but also functional network reorganization^8,9^. Compared with studies on the involvement of the contralateral cortex in remodeling^10,11^, it is still unknown how the contralateral cortex-induced changes in brain networks participate in reorganization.

Network reorganization is dynamic, incessant, and commonly occurs in patients with chronic diseases or brain tumors that progress slower than acute cerebral trauma and stroke^12^. Morphological remodeling and network reorganizing are not bound to the results of functional compensation, and might be an intrinsic alteration after brain lesions appearing^5^. However, accumulating studies have reported that functional plasticity not only depends on morphological remodeling^6,7^ but also functional network reorganization^8,9^. Gliomas originating from the insular lobe are usually diffuse and low-grade^13^. The insular lobe is involved in some resting state networks (such as the salience network and sensorimotor network), and participates in functional modulation^14–16^. Different from the sensorimotor cortex or Broca’s area, the insular lobe is not responsible for detailed functions^17,18^. Furthermore, removal of the entire insular lobe in the lesioned hemisphere does not always cause serious neurologic or neuropsychological impairments^7^. However, this does not mean that the insular lobe is insignificant. The reason for the lack of serious neurological deficits is thought to be related to an increase in contralesional homotopic compensation that frequently increases functional connectivity (FC) in the contralesional hemisphere accompanied with decreases in FC within the ipsilateral damaged network^2,19^. A previous study indicated that diffuse insular low-grade gliomas (DLGGs) cause contralateral hemispheric compensation by increasing the volume of the contralateral insular cortex^11^. This increase in cortical volume provides a basis for network reorganization^20^. Hence, the increase in gray matter volume in the contralesional insular lobe of patients with DLGGs implies that the contralesional insular lobe might join in functional compensation through network reorganization. To our knowledge, no study to date has reported how contralesional insular lobe network reorganization leads to the compensation of damaged functions in DLGG patients.

Graph theoretical analysis is a reliable technique that analyzes topological functional properties using resting state functional MRI (rs-fMRI) in order to identify alterations in functional networks^21,22^. Identifying differences between patients and healthy subjects may help elucidate the altered characteristics of functional networks in various diseases. In this study, we investigated changes in the functional connectivity (FC) and topological properties of the insular lobe in patients with DLGG.

## Results

### Demographic characteristics

In total, 35 right-handed patients with a insular lobe glioma (male, n = 14; age, 42.0 ± 10.8 years) were recruited for this study. All patients were subdivided into the left (insL) and right (insR) groups based on the tumor-affected hemisphere (insL, n = 16). The motor function and muscle strength of all patients was normal. Additionally, 33 healthy participants who were matched for sex, age, and education level were recruited and included in the control group (male, n = 18; all right-handed; age, 38.2 ± 1.5 years). The demographic characteristics of enrolled subjects are shown in Table 1.

**Table 1:** Demographic and clinical characteristics of patient groups.

Demographic and clinical characteristics	InsL (*n* = 16)	InsR (*n* = 19)	Controls (n = 33)	*p* value
**Sex**				
Male	7	7	18	0.48
Female	9	12	15	
Age (years)*	39.0 ± 2.1	43.6 ± 2.4	38.2 ± 1.5	0.12
**Handedness**				–
Right	16	19	33	
Left	0	0	0	
Education level (years)*	12.8 ± 0.9	14.0 ± 0.7	13.4 ± 0.6	0.52
**MMSE scores**				0.28
30	15	14	29	
29	0	4	2	
28	1	1	2	
**Histopathology**				0.72
Astrocytoma	11	11	–	
Oligodendroglioma	5	8	–	
**IDH mutation**				
Mutation	10	13	–	0.71
Wild-type	6	6		
**Chromosome 1p/19q co-deletion**				
Co-deletion	5	8	–	0.72
Non-codeletion	11	11		
**Tumor volume (mL)**	66.27 ± 8.80	66.98 ± 11.80	–	0.96

* Values are presented as means ± standard deviations, unless indicated otherwise.

Using Chi-square test to compare difference of sex distribution among three groups.

Using one-way ANOVA test to compare differences of age and education level among three groups.

Using one-way ANOVA test with nonparametric test to compare the difference of MMSE scores among three groups.

Using Student’s t test to compare difference of tumor volume between insL group and insR group. Using Chi-square test to compare differences of histopathology, IDH mutation, and chromosome 1p/19q co-deletion between the insL and insR groups.

There were no differences in age (p = 0.48), sex (p = 0.12), and educational level (p = 0.52), MMSE scores (p = 0.52) between the patient and control groups. Moreover, There were no differences in histopathology (p = 0.72) and tumor volume (p = 0.96) between the insL and insR groups. Additionally, there were no differences in IDH mutation (p = 0.71) and chromosome 1p/19q co-deletion (p = 0.72) between the patient groups.

### Templates of the healthy hemisphere and whole brain

After excluding tumor-invaded regions and the cerebellum, we extracted templates of the healthy hemisphere and whole brain from the “brainnetome atlas” (including 274 sub-regions). The healthy hemisphere and whole brain templates included 118 and 235 sub-regions, respectively (Supplemental Table 1 and 2).

### Functional connectivity in the healthy hemisphere template

**Figure 1:**
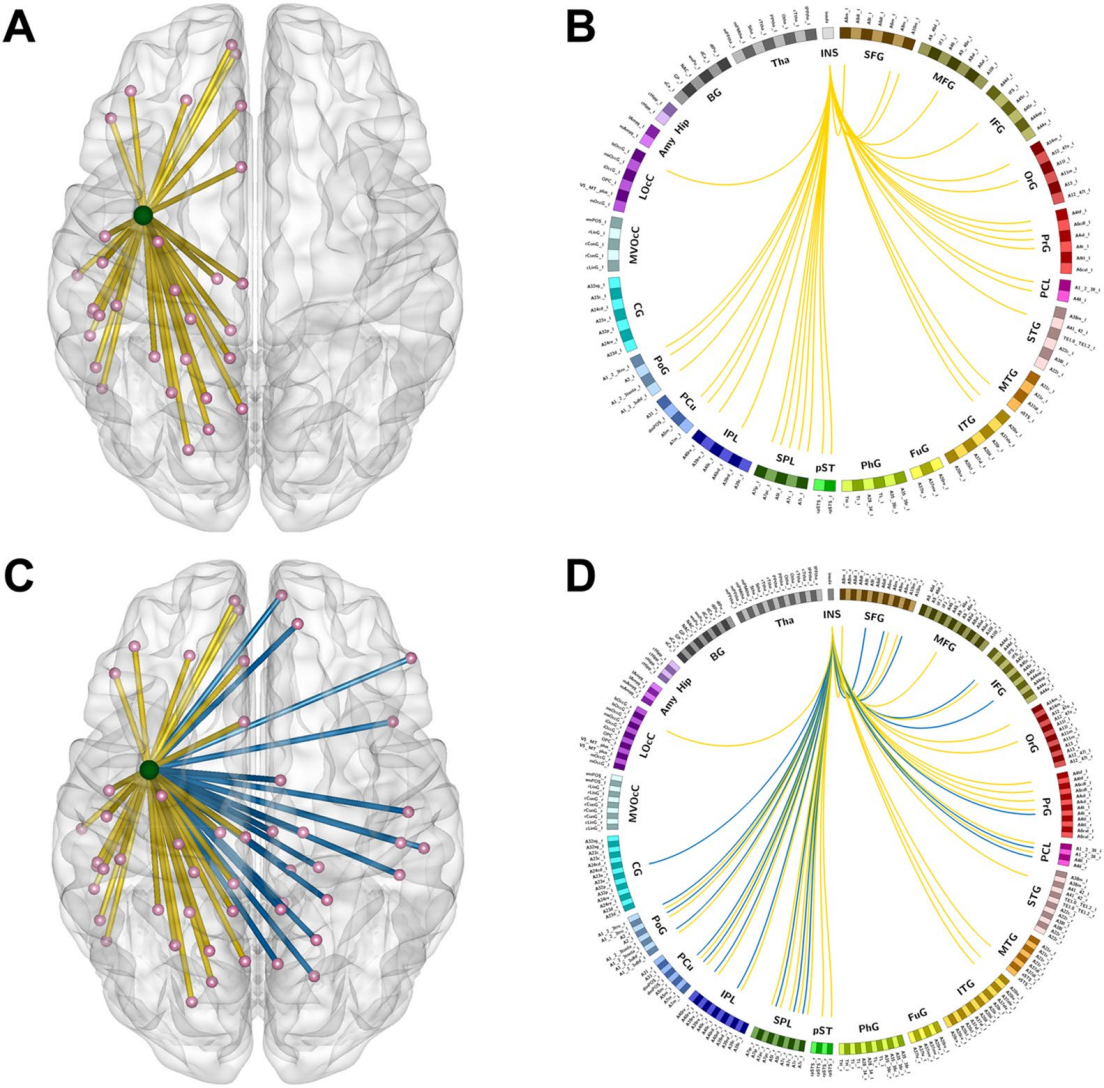
In both, healthy hemisphere and whole brain template, altered functional edges originating from the contralateral insular lobe and functional connectivity in the insR groups were observed compared to those in the healthy group. **(A,B)** Altered functional edges basing on the healthy hemispheric template. **(C,D)** Altered functional edges basing on the whole brain template. Light green node = left insular lobe. The yellow lines present the edges connected left insula to other parts in left hemisphere and blue lines present the edges connected left insula to other parts in right hemisphere.

After NBS comparison, the FC was increased in 33 edges originating from the left insular lobe in the insR group as compared with that in the control group (threshold of p-value = 0.0001, Fig. 1A,B; detailed functional edges are shown in Supplemental Table 3). In addition, there was no difference in the FC of functional edges originating from the right insular lobe between the control and the insL groups (threshold of p-value = 0.0001).

**Table 2:** Topological properties compared between the patient and healthy groups in the healthy template.

	InsL vs healthy (F value)	InsR vs healthy (F value)	InsL vs healthy (threshold of *p* value)	**insR vs healthy (threshold of p value)**
Global efficiency	0.628 ± 0.003 vs 0.638 ± 0.001 (2.20)	0.629 ± 0.002 vs 0.637 ± 0.002 (1.10)	0.020 (0.05)	0.054 (0.05)
Local efficiency	0.817 ± 0.003 vs 0.806 ± 0.002 (2.00)	0.819 ± 0.003 vs 0.804 ± 0.002 (1.31)	0.003 (0.05)	< 0.001 (0.05)
Shortest path length	1.68 ± 0.04 vs 1.60 ± 0.01 (5.33)	1.65 ± 0.01 vs 1.63 ± 0.01 (1.30)	0.019 (0.05)	0.086 (0.05)
Nodal efficiency*	0.62 ± 0.02 vs 0.07 ± 0.01 (1.89)	0.65 ± 0.02 vs 0.67 ± 0.01 (1.01)	0.002 (0.002)	0.431 (0.002)
Nodal local efficiency*	0.82 ± 0.02 vs 0.81 ± 0.01 (3.40)	0.83 ± 0.01 vs 0.82 ± 0.01 (1.01)	0.477 (0.002)	0.251 (0.002)
Nodal shortest path length*	1.67 ± 0.07 vs 1.46 ± 0.03 (3.52)	1.57 ± 0.04 vs 1.53 ± 0.03 (1.04)	0.001 (0.002)	0.443 (0.002)

*The values in this table represent P value of each comparison with False Discovery Rate correction.

### Functional connectivity in whole brain template

After NBS comparison, the FC was increased in 53 edges originating from the left insular lobe in the insR group as compared with that in the control group after NBS correction (threshold of p-value = 0.0001). Among the 53 edges, 20 were connected to the lesioned hemisphere (Fig. 1C,D; detailed functional edges are shown in Supplemental Table 3). Additionally, between the control and the insL groups, no difference was found in the FC of functional edges that originated from the right insular lobe (threshold of p-value = 0.0001).

### Topological properties in the healthy hemisphere template

We observed multiple alterations in the topological properties between the control group and the insL (Fig. S2) and insR (Fig. S3) groups (after FDR correction, detailed information in Table 2). For example, when the tumor grew on the left hemisphere, the global efficiency (p = 0.020) was significantly decreased in the insL group as compared with that in the control group. Additionally, the local efficiency (p = 0.003) and shortest path length (p = 0.019) were significantly increased in the insL group as compared with those in the control group. For the right insular lobe node, nodal efficiency was significantly increased (p = 0.002) and nodal shortest path length was decreased (p = 0.001) as compared with those in the healthy group (p-value threshold after FDR correction was 0.002).

When the tumor grew on the right hemisphere, the local efficiency was significantly increased in the insR group as compared with that in the control group (p < 0.001). However, there were no significant differences in global properties and nodal properties between the insR and healthy groups.

### Topological properties in whole brain template

**Figure 2:**
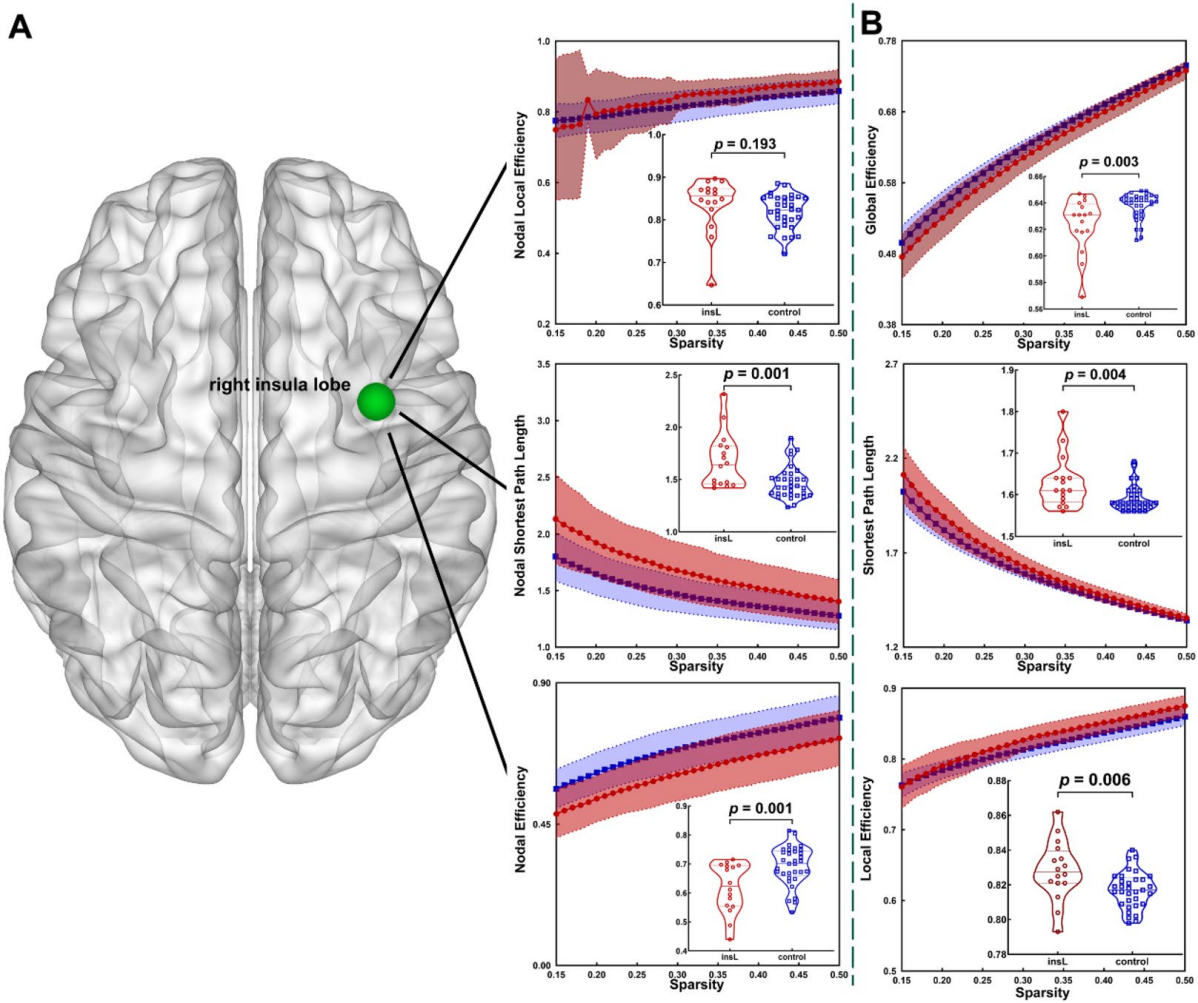
In the whole brain template, compared to the healthy group, altered topological properties were observed in the insL group. **(A)** Altered topological properties at the local level of the insular lobe node. **(B)** Altered topological properties at the global level. Light green node = right insular lobe.

**Figure 3:**
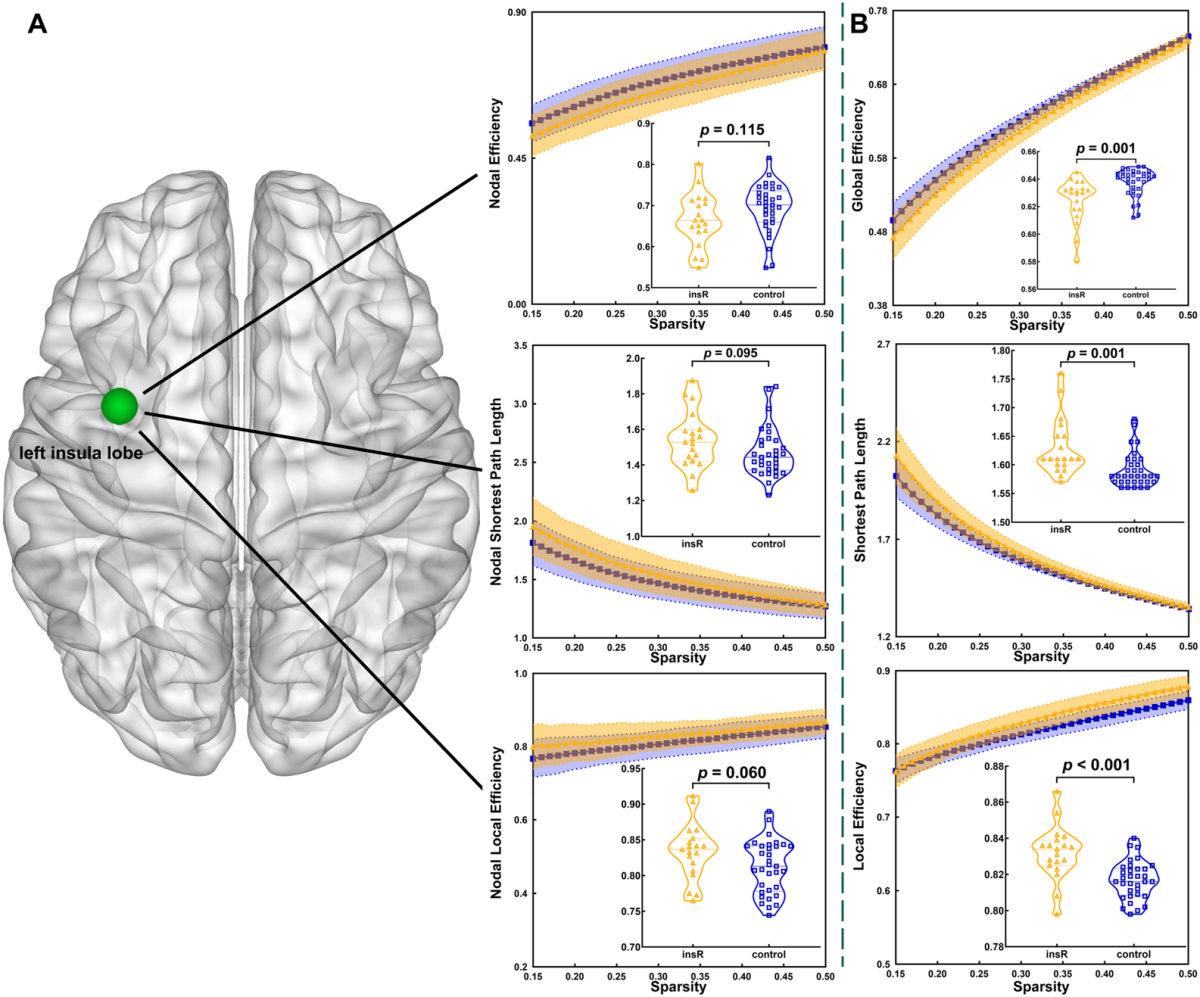
In the whole brain template, compared to the healthy group, altered topological properties were observed in the insR group. **(A)** Altered topological properties at the local level of the insular lobe node. **(B)** Altered topological properties at the global level. Light green node = left insular lobe.

We observed multiple alterations in the topological properties between the control group and the insL (Fig. 2) and insR (Fig. 3) groups (after FDR correction, detailed information in Table 3).

**Table 3:** Topological properties compared between the patient and healthy groups in the whole brain template.

	InsL vs healthy (F value)	InsR vs healthy (F value)	InsL vs healthy (threshold of P value)	**InsR vs healthy (threshold of P value)**
Global efficiency	0.624 ± 0.005 vs 0.638 ± 0.002 (4.33)	0.625 ± 0.004 vs 0.638 ± 0.002 (2.74)	0.003 (0.05)	0.001 (0.05)
Local efficiency	0.828 ± 0.004 vs 0.817 ± 0.002 (2.68)	0.832 ± 0.003 vs 0.817 ± 0.002 (2.04)	0.006 (0.05)	< 0.001 (0.05)
Shortest path length	1.63 ± 0.02 vs 1.59 ± 0.01 (4.34)	1.63 ± 0.01 vs 1.59 ± 0.06 (2.69)	0.004 (0.05)	0.001 (0.05)
Nodal efficiency*	0.62 ± 0.02 vs 0.69 ± 0.01 (1.61)	0.67 ± 0.01 vs 0.69 ± 0.01 (1.21)	0.001 (0.002)	0.115 (0.002)
Nodal local efficiency*	0.84 ± 0.02 vs 0.82 ± 0.01 (2.53)	0.83 ± 0.01 vs 0.81 ± 0.01 (1.10)	0.193 (0.002)	0.060 (0.002)
Nodal shortest path length*	1.68 ± 0.07 vs 1.47 ± 0.03 (2.84)	1.54 ± 0.04 vs 1.47 ± 0.02 (1.34)	0.001 (0.002)	0.095 (0.002)

*The values in this table represent P value of each comparison with False Discovery Rate correction.

When the tumor grew on the left hemisphere, global efficiency was significantly decreased (p = 0.003) in the insL group as compared with that in the control group. Additionally, local efficiency (p = 0.006) and shortest path length (p = 0.004) were significantly increased in the insL group as compared with those in the control group. For the right insular lobe node, nodal efficiency (p = 0.001) was significantly decreased (p-value threshold was 0.002) and nodal shortest path length (p = 0.001) was significantly increased (p-value threshold was 0.002) in the insL group as compared with those in the control group.

When the tumor grew on the right hemisphere, local efficiency (p < 0.001) and shortest path length (p = 0.001) were significantly increased and global efficiency was significantly decreased (p = 0.001) in the insR group as compared with those in the control group. Additionally, no significant alterations in nodal properties between the insR and control groups were detected after FDR correction (p-value threshold was 0.002).

## Discussion

In this study, we investigated the alterations of functional networks in patients with insular DLGGs. Our findings indicate that insular gliomas in different hemispheres can induce different alterations of functional networks, and these alterations may be related to functional compensation^10^.

Rs-fMRI and graph theoretical analysis are widely accepted as reliable methods for investigating alterations in functional networks^23^. Additionally, previous studies have suggested that the alterations in functional networks may be associated with neuroplasticity in DLGG patients. For instance, van Dokkum et al. reported that the right inferior parietal lobe compensated for impaired language function in DLGG patients through the reorganization of attention networks^24^.

The current study investigated the alterations of functional networks in patients with insular gliomas by graph theoretical analysis. The results showed that DLGGs induced functional networks alterations in the whole brain, especially for the mirror insular lobe in the healthy hemisphere. Global efficiency was significantly decreased, and the shortest path length was significantly increased in both the insL and insR groups as compared with those in the control group. These findings are consistent with the findings from previous studies^25–28^, and indicates that the pathways of information conveying change as DLGGs grow.

The alterations of networks that occurred on each side of the insular lobe were different. In the insL group, the nodal shortest path length of the right insular lobe was significantly increased as compared with that in the control group. This finding implies that the pathway of information conveying was prolonged because the edges that originated from the left insular lobe were damaged^21^. Therefore, it can be inferred that when the DLGG originated in the left insular lobe, the right insular lobe replaced the left to convey information through the edges that connected the right insular lobe to some nodes (such as nodes from dorsal prefrontal lobe and parietal lobe) on the lesion hemisphere. Conversely, in the insR group, the FC of some edges originating from the left insular lobe was significantly increased in the insR group as compared with that in the control group, but no significant alterations of the nodal shortest path length and nodal efficiency were found. Most of these functional edges connected the insular lobe to the bilateral precentral gyrus, which is responsible for motor functions^29^; postcentral gyrus, which is responsible for sensory function; inferior parietal lobe, which is responsible for cognitive functions (e.g., calculation, reading, and visual spatial cognition)^30^; and superior and middle temporal gyrus in the left hemisphere, which are responsible for language functions^31^. These findings indicate that when the DLGG grew in the right insular lobe, the networks that were related to left insular lobe altered via strengthening the original functional edges of the whole brain.

Regarding our findings, two potential explanations existed. The one is that contralesional insula participated in functional network reorganizations. Since, the glioma is commonly thought to disrupt functional networks and decrease FC^25,32,33^. Hence, the increasing FC implied that network reorganization occurred similar to what other studies have shown^3,10,19,20^. No significant sensorimotor and abnormal mental status were found in our patients. Thus, our finding might indicate that the damage in these functions might be compensated for through the different network reorganizations. However, the other viewpoint supported that these increasing FC were intrinsic alterations when brain lesions occurred^33^. These atypical increasing FC only reflected a functional adaption in a short-term but not indicated functional compensation^34^. Even some abnormal increasing FCs were related to worse cognitive performance in patients with glioma^35^. Moreover, some studies found that these increasing FC did not ultimately aid in cognitive function^36–38^. Hence, our findings only verified that the contralesional insula participated in brain network alterations. Whether these alterations meant functional network reorganization or functional compensation was controversial.

Furthermore, we propose that these different alterations in insula-related networks are associated with whether DLGGs grew in the dominant hemisphere or not. As is well known, language and other advanced cognitive functions are mainly mediated by the dominant hemisphere^39–42^. Importantly, all enrolled subjects in the current study were right-handed. Accordingly, for those patients, DLGGs involving the left insular lobe were more likely to affect cognitive functions than those involving the right insular lobe^43–48^. Consequently, lesions in the left insular lobe may require greater degrees of alterations of the contralateral insular lobe by building novel functional edges instead of merely strengthening existing ones.

The main limitation of this study was that no effective cognitive tests (such as the Montreal Cognitive Assessment, West Aphasia Battery) were used to evaluate alterations of cognition in glioma patients. Hence, we lacked strong evidence to verify functional plasticity occurring in contralesional insula lobe. In the future, more comprehensive cognitive tests will be applied to validate our findings in future studies. Moreover, histopathology and molecular subtyping may affect the alterations of functional networks in patients with DLGGs. Fortunately, there were no differences in the distributions of histopathology and molecular subtyping of the enrolled patients. However, the small sample size is still a limitation of the study and future large-scale studies are needed to verify our results.

## Methods

The study was approved by the institutional review board of Beijing Tiantan Hospital. Written informed consent was obtained from all study participants. All methods used were carried out in accordance with relevant guidelines and regulations.

### Participants

This study retrospectively reviewed 44 patients who were diagnosed with DLGG in the insular lobe at Beijing Tiantan Hospital between January 2017 and January 2018. The inclusion criteria were as follows: (a) older than 18 years; (b) gliomas growing on the insular lobe; and (c) no history of biopsy, radiotherapy, or chemotherapy. The exclusion criteria were as follows: (a) gliomas involving the bilateral hemispheres, (b) head motion greater than 1° in rotation or 1 mm in translation, and (c) tumor directly invaded other brain lobes (except insula lobe).

### MRI acquisition

All imaging data were acquired with a MAGNETOM Prisma 3 T MR scanner (Siemens, Erlangen, Germany). Parameters of the T1-magnetization prepared rapid acquisition gradient echo were as follow: TR: 2300 ms; TE: 2.3 ms; FOV: 240 × 240 mm^2^; flip angle: 8°; slice number: 192; slice thickness 1.0 mm; voxel size in panel: 1.0 × 1.0 mm^2^). Moreover, the parameters of the T2/FLAIR images were as follow: TR: 5000 ms; TE: 387 ms; FOV: 220 × 220 mm^2^; flip angle: 150°; slice number: 128; thickness: 0.9 mm; and voxel size in panel: 0.4 × 0.4 × 0.9 mm^3^. Additionally, the echo planar imaging sequence for rs-fMRI acquirement was applied (TR: 2000 ms; TE: 30 ms; FOV: 220 × 220 mm^2^; flip angle: 90°; slice number: 30; thickness: 3.0 mm; voxel size in panel: 3.0 × 3.0 mm^2^, acquisition duration: 8 min). Participants were instructed to close their eyes during rs-fMRI acquisition.

### Regions of tumor invasion

The extent of DLGG invasion (shown in Fig. S1) was independently and manually drawn by two neuro-radiologists according to the enhanced regions of the T2/FLAIR images. If the images drawn varied by more than 5%, a third neuro-radiologist with 20 years of experience made the final decision regarding the region location.

### Functional evaluations

All participates underwent a Mini-Mental State Examination (MMSE) to evaluate their mental status before the MRI scan. Moreover, the UK Medical Research Council (MRC) Test for Muscle Strength was conducted to evaluate the basic motor function of all participants.

### Functional MRI preprocessing

The Graph Theoretical Network Analysis (GRETNA) toolbox (https://www.nitrc.org/projects/gretna) in MATLAB (2014a)^49^ was used to preprocess the rs-fMRI data. The pipeline was as follows: (a) transformation of data from DICOM to NIFTI, (b) removal of the first five time points, (c) slice time correction, (d) realignment, (e) spatial normalization (normalized to EPI template)^50^, (f) spatial smoothing (full width half maximum = 4 mm), (g) temporal detrending (linear detrending), (h) regressing out covariance (White matter signal: with WMMask_3mm; CSF signal: with CSFMask_3mm; Head motion: Friston—24 parameters), (i) temporal filtering (0.01–0.08 Hz), and (j) scrubbing (using default parameters and the interpolation strategy: linear interpolation; FD threshold: 0.5; previous time point number: 1; subsequent time point number: 2).

### Regions of interest selection

Regions of interest (ROIs) were extracted from the “brainnetome atlas” (http://www.brainnetome.org/)^51^ to build the FC matrices. The ROI in the healthy hemispheric insular lobe was combined in order to investigate how functional networks compensated. Additionally, two templates (only including the healthy hemisphere and whole brain) were extracted in order to explore how intra-hemispheric and inter-hemispheric functional networks were reorganized.

### Network construction

Pearson correlation coefficients were applied to construct the weighted FC matrix by comparing regional mean time series for all possible pairs of nodes. Consequently, two different sizes of FC matrices were constructed based on the healthy hemisphere template and whole brain template.

### Graph theoretical analysis

To identify the characteristics of topological properties, both negative and positive connections were retained. The weighted FC matrices were transformed into binary FC matrices with absolute values and were processed by GRETNA^21,52^. Both global and nodal topological properties, including the global efficiency, shortest length, local efficiency, nodal global efficiency, nodal local efficiency, and nodal shortest path length, were calculated. All matrices were binarized and absolutized to further analyze the topological properties. Moreover, the graph measurements were normalized using randomized networks (random network number: 10000) during calculation of the topological properties.

### Statistical analysis

Statistical analyses were performed using GraphPad Prism 7 software. Clinical characteristics were compared between the patient and control groups using Student’s t-test, Mann–Whitney U test, one-way ANOVA test, or chi-square test according to the type of data. Additionally, we applied a series of sparsity thresholds (from 0.15 to 0.50, interval 0.01) to explore group differences in network topological properties, and each property was evaluated according to the corresponding matrix. Subsequently, some matrices were generated according to the levels of sparsity. From these generated matrices, the value of each property was calculated. Then, a corresponding curve was made. The area under the curve was applied to compare differences between the patient and control groups.

The differences in FC matrices were assessed using a two-sample t-test between the patient and control groups. Network-based statistics (NBS) were applied to compare differences in FC matrices. The original threshold was equal to 0.001, and the time of permutation was 10,000. The false discovery rate (FDR) correction was applied with an original threshold (p = 0.05) to evaluate differences in nodal topological properties between patient and control groups. Clinical information (age, sex, and education) was used to regress out in statistical comparison.

### Ethical approval

This study was approved by the IRB of Beijing Tiantan Hospital.

### Informed consent

All participants wrote the informed consent of this study.

## Conclusions

Insular DLGGs induced alterations in brain functional networks, and the contralesional insular lobe is a crucial role. Interestingly, when the DLGG grew in the left insular lobe, the right insular lobe replaced the left insular lobe to convey information through the edges that connected the right insular lobe to the same nodes on the lesion hemisphere, and when the DLGG grew in the right insular lobe, the networks were altered via a strengthening of the intrinsic functional edges in the whole brain. In addition, this network alterations were potentially associated with functional network reorganization and functional compensation.

## Supplementary Information

Supplementary Information.

## Data Availability

Anonymized data will be made available on request.
