# Supplementary material for: Contralesional functional network reorganization of the insular cortex in diffuse low-grade glioma patients
Source: Sci Rep. 2021 Jan 12;11:623. doi: 10.1038/s41598-020-79845-3 (PMC7804949; doi:10.1038/s41598-020-79845-3)
Supplement: Supplementary file 1 — Supplementary Information. [file 41598_2020_79845_MOESM1_ESM.docx]

**Title Page**

**Title:** Contralesional functional network reorganization of the insular cortex in diffuse low-grade glioma patients

# Author List:

Shengyu Fang, MD ^1, 2^,

Chunyao Zhou, MD ^1, 2^

Yinyan Wang, MD ^1, 2,^ ^†^

Tao Jiang, MD, PhD ^1, 2, 3,^ ^†^

^1^ Beijing Neurosurgical Institute, Capital Medical University, Beijing, China;

^2^ Department of Neurosurgery, Beijing Tiantan Hospital, Capital Medical University, Beijing, China;

^3^ Research Unit of Accurate Diagnosis, Treatment, and Translational Medicine of Brain Tumors Chinese (2019RU11), Chinese Academy of Medical Sciences

† This author is corresponding author.

# Corresponding Author:

1. Tao Jiang, MD, PhD

Department of Neurosurgery, Beijing Tiantan Hospital, Capital Medical University, 119, the Western Road of the southern 4^th^ Ring Road, Beijing, China.

Postal code: 100070

Tel/Fax: +86-01059976689

E-mail: [taojiang1964@163.com](mailto:taojiang1964@163.com)

2. Yinyan Wang, MD, PhD

Department of Neurosurgery, Beijing Tiantan Hospital, Capital Medical University, 119, the Western Road of the southern 4^th^ Ring Road, Beijing, China.

Postal code: 100070

Tel/Fax: +86-01059976686

E-mail: [tiantanyinyan@126.com](mailto:tiantanyinyan@126.com)

**Keywords:** Glioma; Magnetic resonance imaging; Humans

# Funding Information:

# This work was supported by the Public Welfare Development and Reform Pilot Project of Beijing Medical Research Institute (JYY 2019-5), Beijing Municipal Natural Science Foundation (No. 7202021), and Research Unit of Accurate Diagnosis, Treatment, and Translational Medicine of Brain Tumors Chinese (No. 2019-I2M-5-021).

**Ethical approval：**This study was approved by the IRB of Beijing Tiantan Hospital.

**Informed consent:** All participants wrote the informed consent of this study.

# Supplementary materials

## Part 1. The templates of the healthy hemisphere and the whole brain.

**Supplementary Table 1. Montreal Neurological Institute (MNI) locations of 118 nodes in the template of the healthy hemisphere.**

| **Regions of interesting** | **Modified Cyto-architectonic** | **Tumor in the right hemisphere** | | | **Tumor in the left hemisphere** | | |
| --- | --- | --- | --- | --- | --- | --- | --- |
|  |  | X | Y | Z | X | Y | Z |
| SFG_L(R)_7_1 | medial area BA 8 | -5 | 15 | 54 | 7 | 16 | 54 |
| SFG_L(R)_7_2 | dorsolateral area BA 8 | -18 | 24 | 53 | 22 | 26 | 51 |
| SFG_L(R)_7_3 | lateral area BA 9 | -11 | 49 | 40 | 13 | 48 | 40 |
| SFG_L(R)_7_4 | dorsolateral area BA 6 | -18 | -1 | 65 | 20 | 4 | 64 |
| SFG_L(R)_7_5 | medial area BA 6 | -6 | -5 | 58 | 7 | -4 | 60 |
| SFG_L(R)_7_6 | medial area BA 9 | -5 | 36 | 38 | 6 | 38 | 35 |
| SFG_L(R)_7_7 | medial area BA 10 | -8 | 56 | 15 | 8 | 58 | 13 |
| MFG_L(R)_7_1 | dorsal area BA 9/46 | -27 | 43 | 31 | 30 | 37 | 36 |
| MFG_L(R)_7_2 | inferior frontal junction | -42 | 13 | 36 | 42 | 11 | 39 |
| MFG_L(R)_7_3 | area BA 46 | -28 | 56 | 12 | 28 | 55 | 17 |
| MFG_L(R)_7_4 | ventral area BA 9/46 | -41 | 41 | 16 | 42 | 44 | 14 |
| MFG_L(R)_7_5 | ventrolateral area BA 8 | -33 | 23 | 45 | 42 | 27 | 39 |
| MFG_L(R)_7_6 | ventrolateral area BA 6 | -32 | 4 | 55 | 34 | 8 | 54 |
| MFG_L(R)_7_7 | lateral area BA 10 | -26 | 60 | -6 | 25 | 61 | -4 |
| IFG_L(R)_6_1 | dorsal area BA 44 | -46 | 13 | 24 | 45 | 16 | 25 |
| IFG_L(R)_6_2 | inferior frontal sulcus | -47 | 32 | 14 | 48 | 35 | 13 |
| IFG_L(R)_6_3 | caudal area BA 45 | -53 | 23 | 11 | 54 | 24 | 12 |
| IFG_L(R)_6_4 | rostral area BA 45 | -49 | 36 | -3 | 51 | 36 | -1 |
| IFG_L(R)_6_5 | opercular area BA 44 | -39 | 23 | 4 | 42 | 22 | 3 |
| IFG_L(R)_6_6 | ventral area BA 44 | -52 | 13 | 6 | 54 | 14 | 11 |
| OrG_L(R)_6_1 | medial area BA 14 | -7 | 54 | -7 | 6 | 47 | -7 |
| OrG_L(R)_6_2 | orbital area BA 12/47 | -36 | 33 | -16 | 40 | 39 | -14 |
| OrG_L(R)_6_3 | lateral area BA 11 | -23 | 38 | -18 | 23 | 36 | -18 |
| OrG_L(R)_6_4 | medial area BA 11 | -6 | 52 | -19 | 6 | 57 | -16 |
| OrG_L(R)_6_5 | area BA 13 | -10 | 18 | -19 | 9 | 20 | -19 |
| OrG_L(R)_6_6 | lateral area BA 12/47 | -41 | 32 | -9 | 42 | 31 | -9 |
| PrG_L(R)_6_1 | area BA 4 (head and face region) | -49 | -8 | 39 | 55 | -2 | 33 |
| PrG_L(R)_6_2 | caudal dorsolateral area BA 6 | -32 | -9 | 58 | 33 | -7 | 57 |
| PrG_L(R)_6_3 | area BA 4 (upper limb region) | -26 | -25 | 63 | 34 | -19 | 59 |
| PrG_L(R)_6_4 | area BA 4 (upper limb region) | -13 | -20 | 73 | 15 | -22 | 71 |
| PrG_L(R)_6_5 | area BA 4 (tongue and larynx region) | -52 | 0 | 8 | 54 | 4 | 9 |
| PrG_L(R)_6_6 | caudal ventrolateral area BA 6 | -49 | 5 | 30 | 51 | 7 | 30 |
| PCL_L(R)_2_1 | area BA 1/2/3 (lower limb region) | -8 | -38 | 58 | 10 | -34 | 54 |
| PCL_L(R)_2_2 | area BA 4, (lower limb region) | -4 | -23 | 61 | 5 | -21 | 61 |
| STG_L(R)_6_1 | medial area BA 38 | -32 | 14 | -34 | 31 | 15 | -34 |
| STG_L(R)_6_2 | area BA 41/42 | -54 | -32 | 12 | 54 | -24 | 11 |
| STG_L(R)_6_3 | TE1.0 and TE1.2 | -50 | -11 | 1 | 51 | -4 | -1 |
| STG_L(R)_6_4 | caudal area BA 22 | -62 | -33 | 7 | 66 | -20 | 6 |
| STG_L(R)_6_5 | lateral area BA 38 | -45 | 11 | -20 | 47 | 12 | -20 |
| STG_L(R)_6_6 | rostral area BA 22 | -55 | -3 | -10 | 56 | -12 | -5 |
| MTG_L(R)_4_1 | caudal area BA 21 | -65 | -30 | -12 | 65 | -29 | -13 |
| MTG_L(R)_4_2 | rostral area BA 21 | -53 | 2 | -30 | 51 | 6 | -32 |
| **Regions of interesting** | **Modified Cyto-architectonic** | **Tumor in the right hemisphere** | | | **Tumor in the left hemisphere** | | |
|  |  | X | Y | Z | X | Y | Z |
| MTG_L(R)_4_3 | dorsolateral area BA 37 | -59 | -58 | 4 | 60 | -53 | 3 |
| MTG_L(R)_4_4 | anterior superior temporal sulcus | -58 | -20 | -9 | 58 | -16 | -10 |
| ITG_L(R)_7_1 | intermediate ventral area BA 20 | -45 | -26 | -27 | 46 | -14 | -33 |
| ITG_L(R)_7_2 | extreme lateroventral area BA 37 | -51 | -57 | -15 | 53 | -52 | -18 |
| ITG_L(R)_7_3 | rostral area BA 20 | -43 | -2 | -41 | 40 | 0 | -43 |
| ITG_L(R)_7_4 | intermediate lateral area 20 | -56 | -16 | -28 | 55 | -11 | -32 |
| ITG_L(R)_7_5 | ventrolateral area 37 | -55 | -60 | -6 | 54 | -57 | -8 |
| ITG_L(R)_7_6 | caudal lateral of area BA 20 | -59 | -42 | -16 | 61 | -40 | -17 |
| ITG_L(R)_7_7 | caudal ventral of area BA 20 | -55 | -31 | -27 | 54 | -31 | -26 |
| FuG_L(R)_3_1 | rostral ventral area BA 20 | -33 | -16 | -32 | 33 | -15 | -34 |
| FuG_L(R)_3_2 | medioventral area BA 37 | -31 | -64 | -14 | 31 | -62 | -14 |
| FuG_L(R)_3_3 | lateroventral area BA 37 | -42 | -51 | -17 | 43 | -49 | -19 |
| PhG_L(R)_6_1 | rostral area BA 35/36 | -27 | -7 | -34 | 28 | -8 | -33 |
| PhG_L(R)_6_2 | caudal area BA 35/36 | -25 | -25 | -26 | 26 | -23 | -27 |
| PhG_L(R)_6_3 | posterior para-hippocampal gyrus | -28 | -32 | -18 | 30 | -30 | -18 |
| PhG_L(R)_6_4 | area BA 28/34, entorhinal cortex | -19 | -12 | -30 | 19 | -10 | -30 |
| PhG_L(R)_6_5 | temporal agranular insular cortex | -23 | 2 | -32 | 22 | 1 | -36 |
| PhG_L(R)_6_6 | medial PPHC | -17 | -39 | -10 | 19 | -36 | -11 |
| pSTS_L(R)_2_1 | rostral posterior superior temporal sulcus | -54 | -40 | 4 | 53 | -37 | 3 |
| pSTS_L(R)_2_2 | caudal posterior superior temporal sulcus | -52 | -50 | 11 | 57 | -40 | 12 |
| SPL_L(R)_5_1 | rostral area BA 7 | -16 | -60 | 63 | 9 | -57 | 65 |
| SPL_L(R)_5_2 | caudal area BA 7 | -15 | -71 | 52 | 19 | -69 | 54 |
| SPL_L(R)_5_3 | lateral area BA 5 | -33 | -47 | 50 | 35 | -42 | 54 |
| SPL_L(R)_5_4 | postcentral area BA 7 | -22 | -47 | 65 | 23 | -43 | 67 |
| SPL_L(R)_5_5 | intraparietal area BA 7 (hIP3) | -27 | -59 | 54 | 31 | -54 | 53 |
| IPL_L(R)_6_1 | caudal area BA 39 (PGp) | -34 | -80 | 29 | 45 | -71 | 20 |
| IPL_L(R)_6_2 | rostral dorsal area BA 39 (Hip3) | -38 | -61 | 46 | 39 | -65 | 44 |
| IPL_L(R)_6_3 | rostral dorsal area BA 40 (PFt) | -51 | -33 | 42 | 47 | -35 | 45 |
| IPL_L(R)_6_4 | caudal area BA 40 (PFm) | -56 | -49 | 38 | 57 | -44 | 38 |
| IPL_L(R)_6_5 | rostroventral area BA 39 (PGa) | -47 | -65 | 26 | 53 | -54 | 25 |
| IPL_L(R)_6_6 | rostroventral area BA 40 (PFop) | -53 | -31 | 23 | 55 | -26 | 26 |
| PCun_L(R)_4_1 | medial area BA 7 (PEp) | -5 | -63 | 51 | 6 | -65 | 51 |
| PCun_L(R)_4_2 | medial area BA 5 (PEm) | -8 | -47 | 57 | 7 | -47 | 58 |
| PCun_L(R)_4_3 | dorsomedial parietal occipital sulcus | -12 | -67 | 25 | 16 | -64 | 25 |
| PCun_L(R)_4_4 | area BA 31 (Lc1) | -6 | -55 | 34 | 6 | -54 | 35 |
| PoG_L(R)_4_1 | BA 1/2/3 (limb, head and face region) | -50 | -16 | 43 | 50 | -14 | 44 |
| PoG_L(R)_4_2 | BA 1/2/3 (tongue and larynx region) | -56 | -14 | 16 | 56 | -10 | 15 |
| PoG_L(R)_4_3 | area BA 2 | -46 | -30 | 50 | 48 | -24 | 48 |
| PoG_L(R)_4_4 | area BA 1/2/3 (trunk region) | -21 | -35 | 68 | 20 | -33 | 69 |
| CG_L(R)_7_1 | dorsal area BA 23 | -4 | -39 | 31 | 4 | -37 | 32 |
| CG_L(R)_7_2 | rostroventral area BA 24 | -3 | 8 | 25 | 5 | 22 | 12 |
| CG_L(R)_7_3 | pre-genual area BA 32 | -6 | 34 | 21 | 5 | 28 | 27 |
| CG_L(R)_7_4 | ventral area BA 23 | -8 | -47 | 10 | 9 | -44 | 11 |
| CG_L(R)_7_5 | caudal dorsal area BA 24 | -5 | 7 | 37 | 4 | 6 | 38 |
| CG_L(R)_7_6 | caudal area BA 23 | -7 | -23 | 41 | 6 | -20 | 40 |
| CG_L(R)_7_7 | sub-genual area BA 32 | -4 | 39 | -2 | 5 | 41 | 6 |
| MVOcC_L(R)_5_1 | caudal lingual gyrus | -11 | -82 | -11 | 10 | -85 | -9 |
| MVOcC_L(R)_5_2 | rostral cuneus gyrus | -5 | -81 | 10 | 7 | -76 | 11 |
| MVOcC_L(R)_5_3 | caudal cuneus gyrus | -6 | 94 | 1 | 8 | -90 | 12 |
| MVOcC_L(R)_5_4 | rostral lingual gyrus | -17 | -60 | -6 | 18 | -60 | -7 |
| **Regions of interesting** | **Modified Cyto-architectonic** | **Tumor in the right hemisphere** | | | **Tumor in the left hemisphere** | | |
|  |  | X | Y | Z | X | Y | Z |
| MVOcC_L(R)_5_5 | ventromedial parietal occipital sulcus | -13 | -68 | 12 | 15 | -63 | 12 |
| LOcC_L(R)_4_1 | middle occipital gyrus | -31 | -89 | 11 | 34 | -86 | 11 |
| LOcC_L(R)_4_2 | area V5/MT+ | -46 | -74 | 3 | 48 | -70 | -1 |
| LOcC_L(R)_4_3 | occipital polar cortex | -18 | -99 | 2 | 22 | -97 | 4 |
| LOcC_L(R)_4_4 | inferior occipital gyrus | -30 | -88 | -12 | 32 | -85 | -12 |
| LOcC_L(R)_2_1 | medial superior occipital gyrus | -11 | -88 | 31 | 16 | -85 | 34 |
| LOcC_L(R)_2_2 | lateral superior occipital gyrus | -22 | -77 | 36 | 29 | -75 | 36 |
| Amyg_L(R)_2_1 | medial amygdala | -19 | -2 | -20 | 19 | -2 | -19 |
| Amyg_L(R)_2_2 | lateral amygdala | -27 | -4 | -20 | 28 | -3 | -20 |
| Hipp_L(R)_2_1 | rostral hippocampus | -22 | -14 | -19 | 22 | -12 | -20 |
| Hipp_L(R)_2_2 | caudal hippocampus | -28 | -30 | -10 | 29 | -27 | -10 |
| BG_L(R)_6_1 | ventral caudate | -12 | 14 | 0 | 15 | 14 | -2 |
| BG_L(R)_6_2 | globus pallidus | -22 | 2 | 4 | 22 | -2 | 3 |
| BG_L(R)_6_3 | nucleus accumbens | -17 | 3 | -9 | 15 | 8 | -9 |
| BG_L(R)_6_4 | ventromedial putamen | -23 | 7 | -4 | 22 | 8 | -1 |
| BG_L(R)_6_5 | dorsal caudate | -14 | 2 | 16 | 14 | 5 | 14 |
| BG_L(R)_6_6 | dorsolateral putamen | -28 | -5 | 2 | 29 | -3 | 1 |
| Tha_L(R)_8_1 | medial pre-frontal thalamus | -7 | -12 | 5 | 7 | -11 | 6 |
| Tha_L(R)_8_2 | pre-motor thalamus | -18 | -13 | 3 | 12 | -14 | 1 |
| Tha_L(R)_8_3 | sensory thalamus | -18 | -23 | 4 | 18 | -22 | 3 |
| Tha_L(R)_8_4 | rostral temporal thalamus | -7 | -14 | 7 | 3 | -13 | 5 |
| Tha_L(R)_8_5 | posterior parietal thalamus | -16 | -24 | 6 | 15 | -25 | 6 |
| Tha_L(R)_8_6 | occipital thalamus | -15 | -28 | 4 | 13 | -27 | 8 |
| Tha_L(R)_8_7 | caudal temporal thalamus | -12 | -22 | 13 | 10 | -14 | 14 |
| Tha_L(R)_8_8 | lateral pre-frontal thalamus | -11 | -14 | 2 | 13 | -16 | 7 |
| insL(R) | insula lobe in healthy hemisphere | -36 | -1 | 1 | 37 | 1 | 0 |

* BA = Brodmann area.

**Supplementary Table 2. Montreal Neurological Institute (MNI) locations of 235 nodes in the template of the whole brain.**

| **Regions of interesting** | **Modified Cyto-architectonic** | **Template coordinates** | | |
| --- | --- | --- | --- | --- |
|  |  | X | Y | Z |
| SFG_L_7_1 | medial area BA 8 | -5 | 15 | 54 |
| SFG_R_7_1 |  | 7 | 16 | 54 |
| SFG_L_7_2 | dorsolateral area BA 8 | -18 | 24 | 53 |
| SFG_R_7_2 |  | 22 | 26 | 51 |
| SFG_L_7_3 | lateral area BA 9 | -11 | 49 | 40 |
| SFG_R_7_3 |  | 13 | 48 | 40 |
| SFG_L_7_4 | dorsolateral area BA 6 | -18 | -1 | 65 |
| SFG_R_7_4 |  | 20 | 4 | 64 |
| SFG_L_7_5 | medial area BA 6 | -6 | -5 | 58 |
| SFG_R_7_5 |  | 7 | -4 | 60 |
| SFG_L_7_6 | medial area BA 9 | -5 | 36 | 38 |
| SFG_R_7_6 |  | 6 | 38 | 35 |
| SFG_L_7_7 | medial area BA 10 | -8 | 56 | 15 |
| SFG_R_7_7 |  | 8 | 58 | 13 |
| MFG_L_7_1 | dorsal area BA 9/46 | -27 | 43 | 31 |
| MFG_R_7_1 |  | 30 | 37 | 36 |
| MFG_L_7_2 | inferior frontal junction | -42 | 13 | 36 |
| MFG_R_7_2 |  | 42 | 11 | 39 |
| MFG_L_7_3 | area BA 46 | -28 | 56 | 12 |
| MFG_R_7_3 |  | 28 | 55 | 17 |
| MFG_L_7_4 | ventral area BA 9/46 | -41 | 41 | 16 |
| MFG_R_7_4 |  | 42 | 44 | 14 |
| MFG_L_7_5 | ventrolateral area BA 8 | -33 | 23 | 45 |
| MFG_R_7_5 |  | 42 | 27 | 39 |
| MFG_L_7_6 | ventrolateral area BA 6 | -32 | 4 | 55 |
| MFG_R_7_6 |  | 34 | 8 | 54 |
| MFG_L_7_7 | lateral area BA 10 | -26 | 60 | -6 |
| MFG_R_7_7 |  | 25 | 61 | -4 |
| IFG_L_6_1 | dorsal area BA 44 | -46 | 13 | 24 |
| IFG_R_6_1 |  | 45 | 16 | 25 |
| IFG_L_6_2 | inferior frontal sulcus | -47 | 32 | 14 |
| IFG_R_6_2 |  | 48 | 35 | 13 |
| IFG_L_6_3 | caudal area BA 45 | -53 | 23 | 11 |
| IFG_R_6_3 |  | 54 | 24 | 12 |
| IFG_L_6_4 | rostral area BA 45 | -49 | 36 | -3 |
| IFG_R_6_4 |  | 51 | 36 | -1 |
| IFG_L_6_5 | opercular area BA 44 | -39 | 23 | 4 |
| IFG_R_6_5 |  | 42 | 22 | 3 |
| IFG_L_6_6 | ventral area BA 44 | -52 | 13 | 6 |
| IFG_R_6_6 |  | 54 | 14 | 11 |
| OrG_L_6_1 | medial area BA 14 | -7 | 54 | -7 |
| OrG_R_6_1 |  | 6 | 47 | -7 |
| OrG_L_6_2 | orbital area BA 12/47 | -36 | 33 | -16 |
| OrG_R_6_2 |  | 40 | 39 | -14 |
| OrG_L_6_3 | lateral area BA 11 | -23 | 38 | -18 |
| OrG_R_6_3 |  | 23 | 36 | -18 |
| OrG_L_6_4 | medial area BA 11 | -6 | 52 | -19 |
| OrG_R_6_4 |  | 6 | 57 | -16 |
| OrG_L_6_5 | area BA 13 | -10 | 18 | -19 |
| OrG_R_6_5 |  | 9 | 20 | -19 |
| **Regions of interesting** | **Modified Cyto-architectonic** | **Template coordinates** | | |
|  |  | X | Y | Z |
| OrG_L_6_6 | lateral area BA 12/47 | -41 | 32 | -9 |
| OrG_R_6_6 |  | 42 | 31 | -9 |
| PrG_L_6_1 | area BA 4 (head and face region) | -49 | -8 | 39 |
| PrG_R_6_1 |  | 55 | -2 | 33 |
| PrG_L_6_2 | caudal dorsolateral area 6 | -32 | -9 | 58 |
| PrG_R_6_2 |  | 33 | -7 | 57 |
| PrG_L_6_3 | area BA 4 (upper limb region) | -26 | -25 | 63 |
| PrG_R_6_3 |  | 34 | -19 | 59 |
| PrG_L_6_4 | area BA 4 (upper limb region) | -13 | -20 | 73 |
| PrG_R_6_4 |  | 15 | -22 | 71 |
| PrG_L_6_5 | area BA 4 (tongue and larynx region) | -52 | 0 | 8 |
| PrG_R_6_5 |  | 54 | 4 | 9 |
| PrG_L_6_6 | caudal ventrolateral area BA 6 | -49 | 5 | 30 |
| PrG_R_6_6 |  | 51 | 7 | 30 |
| PCL_L_2_1 | area BA 1/2/3 (lower limb region) | -8 | -38 | 58 |
| PCL_R_2_1 |  | 10 | -34 | 54 |
| PCL_L_2_2 | area BA 4 (lower limb region) | -4 | -23 | 61 |
| PCL_R_2_2 |  | 5 | -21 | 61 |
| STG_L_6_1 | medial area BA 38 | -32 | 14 | -34 |
| STG_R_6_1 |  | 31 | 15 | -34 |
| STG_L_6_2 | area BA 41/42 | -54 | -32 | 12 |
| STG_R_6_2 |  | 54 | -24 | 11 |
| STG_L_6_3 | TE1.0 and TE1.2 | -50 | -11 | 1 |
| STG_R_6_3 |  | 51 | -4 | -1 |
| STG_L_6_4 | caudal area BA 22 | -62 | -33 | 7 |
| STG_R_6_4 |  | 66 | -20 | 6 |
| STG_L_6_5 | lateral area BA 38 | -45 | 11 | -20 |
| STG_R_6_5 |  | 47 | 12 | -20 |
| STG_L_6_6 | rostral area BA 22 | -55 | -3 | -10 |
| STG_R_6_6 |  | 56 | -12 | -5 |
| MTG_L_4_1 | caudal area BA 21 | -65 | -30 | -12 |
| MTG_R_4_1 |  | 65 | -29 | -13 |
| MTG_L_4_2 | rostral area BA 21 | -53 | 2 | -30 |
| MTG_R_4_2 |  | 51 | 6 | -32 |
| MTG_L_4_3 | dorsolateral area BA 37 | -59 | -58 | 4 |
| MTG_R_4_3 |  | 60 | -53 | 3 |
| MTG_L_4_4 | anterior superior temporal sulcus | -58 | -20 | -9 |
| MTG_R_4_4 |  | 58 | -16 | -10 |
| ITG_L_7_1 | intermediate ventral area BA 20 | -45 | -26 | -27 |
| ITG_R_7_1 |  | 46 | -14 | -33 |
| ITG_L_7_2 | extreme lateroventral area BA 37 | -51 | -57 | -15 |
| ITG_R_7_2 |  | 53 | -52 | -18 |
| ITG_L_7_3 | rostral area BA 20 | -43 | -2 | -41 |
| ITG_R_7_3 |  | 40 | 0 | -43 |
| ITG_L_7_4 | intermediate lateral area BA 20 | -56 | -16 | -28 |
| ITG_R_7_4 |  | 55 | -11 | -32 |
| ITG_L_7_5 | ventrolateral area BA 37 | -55 | -60 | -6 |
| ITG_R_7_5 |  | 54 | -57 | -8 |
| ITG_L_7_6 | caudal lateral of area BA 20 | -59 | -42 | -16 |
| ITG_R_7_6 |  | 61 | -40 | -17 |
| ITG_L_7_7 | caudal ventral of area BA 20 | -55 | -31 | -27 |
| ITG_R_7_7 |  | 54 | -31 | -26 |
| FuG_L_3_1 | rostral ventral area BA 20 | -33 | -16 | -32 |
| FuG_R_3_1 |  | 33 | -15 | -34 |
| **Regions of interesting** | **Modified Cyto-architectonic** | **Template coordinates** | | |
|  |  | X | Y | Z |
| FuG_L_3_2 | medioventral area BA 37 | -31 | -64 | -14 |
| FuG_R_3_2 |  | 31 | -62 | -14 |
| FuG_L_3_3 | lateroventral area BA 37 | -42 | -51 | -17 |
| FuG_R_3_3 |  | 43 | -49 | -19 |
| PhG_L_6_1 | rostral area BA 35/36 | -27 | -7 | -34 |
| PhG_R_6_1 |  | 28 | -8 | -33 |
| PhG_L_6_2 | caudal area BA 35/36 | -25 | -25 | -26 |
| PhG_R_6_2 |  | 26 | -23 | -27 |
| PhG_L_6_3 | posterior para-hippocampal gyrus | -28 | -32 | -18 |
| PhG_R_6_3 |  | 30 | -30 | -18 |
| PhG_L_6_4 | area BA 28/34, entorhinal cortex | -19 | -12 | -30 |
| PhG_R_6_4 |  | 19 | -10 | -30 |
| PhG_L_6_5 | temporal agranular insular cortex | -23 | 2 | -32 |
| PhG_R_6_5 |  | 22 | 1 | -36 |
| PhG_L_6_6 | medial PPHC | -17 | -39 | -10 |
| PhG_R_6_6 |  | 19 | -36 | -11 |
| pSTS_L_2_1 | rostral posterior superior temporal sulcus | -54 | -40 | 4 |
| pSTS_R_2_1 |  | 53 | -37 | 3 |
| pSTS_L_2_2 | caudal posterior superior temporal sulcus | -52 | -50 | 11 |
| pSTS_R_2_2 |  | 57 | -40 | 12 |
| SPL_L_5_1 | rostral area BA 7 | -16 | -60 | 63 |
| SPL_R_5_1 |  | 9 | -57 | 65 |
| SPL_L_5_2 | caudal area BA 7 | -15 | -71 | 52 |
| SPL_R_5_2 |  | 19 | -69 | 54 |
| SPL_L_5_3 | lateral area BA 5 | -33 | -47 | 50 |
| SPL_R_5_3 |  | 35 | -42 | 54 |
| SPL_L_5_4 | postcentral area BA 7 | -22 | -47 | 65 |
| SPL_R_5_4 |  | 23 | -43 | 67 |
| SPL_L_5_5 | intraparietal area BA 7 (hIP3) | -27 | -59 | 54 |
| SPL_R_5_5 |  | 31 | -54 | 53 |
| IPL_L_6_1 | caudal area BA 39 (PGp) | -34 | -80 | 29 |
| IPL_R_6_1 |  | 45 | -71 | 20 |
| IPL_L_6_2 | rostral dorsal area BA 39 (Hip3) | -38 | -61 | 46 |
| IPL_R_6_2 |  | 39 | -65 | 44 |
| IPL_L_6_3 | rostral dorsal area BA 40 (PFt) | -51 | -33 | 42 |
| IPL_R_6_3 |  | 47 | -35 | 45 |
| IPL_L_6_4 | caudal area BA 40 (PFm) | -56 | -49 | 38 |
| IPL_R_6_4 |  | 57 | -44 | 38 |
| IPL_L_6_5 | rostroventral area BA 39 (PGa) | -47 | -65 | 26 |
| IPL_R_6_5 |  | 53 | -54 | 25 |
| IPL_L_6_6 | rostroventral area BA 40 (PFop) | -53 | -31 | 23 |
| IPL_R_6_6 |  | 55 | -26 | 26 |
| PCun_L_4_1 | medial area BA 7 (PEp) | -5 | -63 | 51 |
| PCun_R_4_1 |  | 6 | -65 | 51 |
| PCun_L_4_2 | medial area BA 5 (PEm) | -8 | -47 | 57 |
| PCun_R_4_2 |  | 7 | -47 | 58 |
| PCun_L_4_3 | dorsomedial parietal occipital sulcus | -12 | -67 | 25 |
| PCun_R_4_3 |  | 16 | -64 | 25 |
| PCun_L_4_4 | area BA 31 (Lc1) | -6 | -55 | 34 |
| PCun_R_4_4 |  | 6 | -54 | 35 |
| PoG_L_4_1 | area BA 1/2/3 (limb, head and face region) | -50 | -16 | 43 |
| PoG_R_4_1 |  | 50 | -14 | 44 |
| PoG_L_4_2 | area BA 1/2/3 (tongue and larynx region) | -56 | -14 | 16 |
| PoG_R_4_2 |  | 56 | -10 | 15 |
| **Regions of interesting** | **Modified Cyto-architectonic** | **Template coordinates** | | |
|  |  | X | Y | Z |
| PoG_L_4_3 | area BA 2 | -46 | -30 | 50 |
| PoG_R_4_3 |  | 48 | -24 | 48 |
| PoG_L_4_4 | area BA 1/2/3 (trunk region) | -21 | -35 | 68 |
| PoG_R_4_4 |  | 20 | -33 | 69 |
| CG_L_7_1 | dorsal area BA 23 | -4 | -39 | 31 |
| CG_R_7_1 |  | 4 | -37 | 32 |
| CG_L_7_2 | rostroventral area BA 24 | -3 | 8 | 25 |
| CG_R_7_2 |  | 5 | 22 | 12 |
| CG_L_7_3 | pre-genual area BA 32 | -6 | 34 | 21 |
| CG_R_7_3 |  | 5 | 28 | 27 |
| CG_L_7_4 | ventral area BA 23 | -8 | -47 | 10 |
| CG_R_7_4 |  | 9 | -44 | 11 |
| CG_L_7_5 | caudal dorsal area BA 24 | -5 | 7 | 37 |
| CG_R_7_5 |  | 4 | 6 | 38 |
| CG_L_7_6 | caudal area BA 23 | -7 | -23 | 41 |
| CG_R_7_6 |  | 6 | -20 | 40 |
| CG_L_7_7 | sub-genual area BA 32 | -4 | 39 | -2 |
| CG_R_7_7 |  | 5 | 41 | 6 |
| MVOcC_L_5_1 | caudal lingual gyrus | -11 | -82 | -11 |
| MVOcC_R_5_1 |  | 10 | -85 | -9 |
| MVOcC_L_5_2 | rostral cuneus gyrus | -5 | -81 | 10 |
| MVOcC_R_5_2 |  | 7 | -76 | 11 |
| MVOcC_L_5_3 | caudal cuneus gyrus | -6 | 94 | 1 |
| MVOcC_R_5_3 |  | 8 | -90 | 12 |
| MVOcC_L_5_4 | rostral lingual gyrus | -17 | -60 | -6 |
| MVOcC_R_5_4 |  | 18 | -60 | -7 |
| MVOcC_L_5_5 | ventromedial parietal occipital sulcus | -13 | -68 | 12 |
| MVOcC_R_5_5 |  | 15 | -63 | 12 |
| LOcC_L_4_1 | middle occipital gyrus | -31 | -89 | 11 |
| LOcC_R_4_1 |  | 34 | -86 | 11 |
| LOcC_L_4_2 | area V5/MT+ | -46 | -74 | 3 |
| LOcC_R_4_2 |  | 48 | -70 | -1 |
| LOcC_L_4_3 | occipital polar cortex | -18 | -99 | 2 |
| LOcC_R_4_3 |  | 22 | -97 | 4 |
| LOcC_L_4_4 | inferior occipital gyrus | -30 | -88 | -12 |
| LOcC_R_4_4 |  | 32 | -85 | -12 |
| LOcC_L_2_1 | medial superior occipital gyrus | -11 | -88 | 31 |
| LOcC_R_2_1 |  | 16 | -85 | 34 |
| LOcC_L_2_2 | lateral superior occipital gyrus | -22 | -77 | 36 |
| LOcC_R_2_2 |  | 29 | -75 | 36 |
| Amyg_L_2_1 | medial amygdala | -19 | -2 | -20 |
| Amyg_R_2_1 |  | 19 | -2 | -19 |
| Amyg_L_2_2 | lateral amygdala | -27 | -4 | -20 |
| Amyg_R_2_2 |  | 28 | -3 | -20 |
| Hipp_L_2_1 | rostral hippocampus | -22 | -14 | -19 |
| Hipp_R_2_1 |  | 22 | -12 | -20 |
| Hipp_L_2_2 | caudal hippocampus | -28 | -30 | -10 |
| Hipp_R_2_2 |  | 29 | -27 | -10 |
| BG_L_6_1 | ventral caudate | -12 | 14 | 0 |
| BG_R_6_1 |  | 15 | 14 | -2 |
| BG_L_6_2 | globus pallidus | -22 | 2 | 4 |
| BG_R_6_2 |  | 22 | -2 | 3 |
| BG_L_6_3 | nucleus accumbens | -17 | 3 | -9 |
| BG_R_6_3 |  | 15 | 8 | -9 |
| **Regions of interesting** | **Modified Cyto-architectonic** | **Template coordinates** | | |
|  |  | X | Y | Z |
| BG_L_6_4 | ventromedial putamen | -23 | 7 | -4 |
| BG_R_6_4 |  | 22 | 8 | -1 |
| BG_L_6_5 | dorsal caudate | -14 | 2 | 16 |
| BG_R_6_5 |  | 14 | 5 | 14 |
| BG_L_6_6 | dorsolateral putamen | -28 | -5 | 2 |
| BG_R_6_6 |  | 29 | -3 | 1 |
| Tha_L_8_1 | medial pre-frontal thalamus | -7 | -12 | 5 |
| Tha_R_8_1 |  | 7 | -11 | 6 |
| Tha_L_8_2 | pre-motor thalamus | -18 | -13 | 3 |
| Tha_R_8_2 |  | 12 | -14 | 1 |
| Tha_L_8_3 | sensory thalamus | -18 | -23 | 4 |
| Tha_R_8_3 |  | 18 | -22 | 3 |
| Tha_L_8_4 | rostral temporal thalamus | -7 | -14 | 7 |
| Tha_R_8_4 |  | 3 | -13 | 5 |
| Tha_L_8_5 | posterior parietal thalamus | -16 | -24 | 6 |
| Tha_R_8_5 |  | 15 | -25 | 6 |
| Tha_L_8_6 | occipital thalamus | -15 | -28 | 4 |
| Tha_R_8_6 |  | 13 | -27 | 8 |
| Tha_L_8_7 | caudal temporal thalamus | -12 | -22 | 13 |
| Tha_R_8_7 |  | 10 | -14 | 14 |
| Tha_L_8_8 | lateral pre-frontal thalamus | -11 | -14 | 2 |
| Tha_R_8_8 |  | 13 | -16 | 7 |
| insL OR insR | left insula lobe OR right insula lobe | 37  (-36) | 1  (-1) | 0  (1) |

* BA = Brodmann area.

## Part 2. The specific functional edges originating from the healthy insula lobe and increasing the functional connectivity

### Supplementary Table 3. The alterations of functional edges in insR group within the whole brain template

| **Functional edges from right insula lobe** | **Modified Cyto-architectonic** | **Functional connectivity**  **(mean ± SEM)** | | **p Value** |
| --- | --- | --- | --- | --- |
|  |  | **insR group** | **healthy group** |  |
| A8m_l | medial area BA 8, L | 0.940 ± 0.079 | 0.631 ± 0.045 | 3.67e-05 |
| A9l_r | lateral area BA 9, R | 0.422 ± 0.078 | 0.146 ± 0.040 | 3.58e-05 |
| A6m_r | medial area BA 6, R | 1.065 ± 0.063 | 0.775 ± 0.051 | 3.51e-05 |
| A9m_l | medial area BA 9, L | 0.667 ± 0.075 | 0.376 ± 0.042 | 3.69e-05 |
| A10m_l | medial area BA 10, L | 0.572 ± 0.095 | 0.243 ± 0.034 | 3.89e-05 |
| A10m_r | medial area BA 10, R | 0.507 ± 0.0805 | 0.220 ± 0.038 | 3.66e-05 |
| A9_46v_l | ventral area BA 9/46,L | 0.780 ± 0.059 | 0.478 ± 0.044 | 4.13e-05 |
| A8vl_l | ventrolateral area BA 8, L | 0.455 ± 0.084 | 0.159 ± 0.045 | 3.61e-05 |
| A8vl_r | ventrolateral area BA 8, R | 0.469 ± 0.063 | 0.235 ± 0.042 | 3.57e-05 |
| A44d_r | dorsal area BA 44, R | 0.677 ± 0.079 | 0.397 ± 0.037 | 3.89e-05 |
| IFS_l | Inferior frontal sulcus, L | 0.690 ± 0.061 | 0.435 ± 0.043 | 4.31e-05 |
| A45r_r | rostral area BA 45, R | 0.812 ± 0.059 | 0.554 ± 0.037 | 3.54e-05 |
| A14m_l | medial area BA 14, L | 0.475 ± 0.069 | 0.170 ± 0.033 | 3.63e-05 |
| A11l_l | lateral area BA 11, L | 0.766 ± 0.082 | 0.470 ± 0.043 | 3.68e-05 |
| A4hf_l | area BA 4 (head and face region), L | 0.828 ± 0.080 | 0.533 ± 0.042 | 3.90e-05 |
| A6cdl_l | caudal dorsolateral area BA 6, L | 0.835 ± 0.065 | 0.523 ± 0.052 | 4.47e-05 |
| A4ul_l | area BA 4 (upper limb region), L | 0.721 ± 0.072 | 0.398 ± 0.047 | 4.16e-05 |
| A4t_l | area BA 4 (trunk region), L | 0.748 ± 0.071 | 0.421 ± 0.038 | 4.99e-05 |
| A4t_r | area BA 4 (trunk region), R | 0.782 ± 0.073 | 0.432 ± 0.048 | 3.70e-05 |
| A1_2_3ll_l | area BA 1/2/3 (lower limb region), L | 0.855 ± 0.063 | 0.508 ± 0.038 | 3.80e-05 |
| A1_2_3ll_r | area BA 1/2/3 (lower limb region), R | 0.926 ± 0.056 | 0.638 ± 0.049 | 4.31e-05 |
| A4ll_l | area BA 4 (lower limb region), L | 0.829 ± 0.079 | 0.501 ± 0.048 | 6.19e-05 |
| A4ll_r | area BA 4 (lower limb region), R | 0.878 ± 0.079 | 0.503 ± 0.048 | 3.51e-05 |
| A41_42_l | area BA 41/42, L | 1.250 ± 0.056 | 0.873 ± 0.034 | 3.61e-05 |
| aSTS_l | anterior superior temporal sulcus, L | 0.635 ± 0.089 | 0.313 ± 0.047 | 4.60e-05 |
| A20iv_l | intermediate ventral area BA 20, L | 0.657 ± 0.069 | 0.372 ± 0.045 | 3.75e-05 |
| A37elv_l | extreme lateroventral area BA 37, L | 0.721 ± 0.066 | 0.349 ± 0.042 | 3.67e-05 |
| rpSTS_l | rostro-posterior superior temporal sulcus, L | 0.776 ± 0.090 | 0.453 ± 0.040 | 3.58e-05 |
| **Functional edges from right insula lobe** | **Modified Cyto-architectonic** | **Functional connectivity**  **(mean ± SEM)** | | **p Value** |
|  |  | **insL group** | **healthy group** |  |
| cpSTS_l | caudo-posterior superior temporal sulcus, L | 0.823 ± 0.068 | 0.496 ± 0.047 | 4.06e-05 |
| A7r_l | rostral area BA 7, L | 0.675 ± 0.053 | 0.359 ± 0.041 | 4.54e-05 |
| A7r_r | rostral area BA 7, R | 0.699 ± 0.054 | 0.377 ± 0.045 | 4.33e-05 |
| A7c_l | caudal area BA 7, L | 0.617 ± 0.058 | 0.309 ± 0.040 | 4.47e-05 |
| A7c_r | caudal area BA 7, R | 0.628 ± 0.057 | 0.326 ± 0.041 | 4.36e-05 |
| A5l_l | lateral area BA 5, L | 0.751 ± 0.059 | 0.393 ± 0.048 | 4.62e-05 |
| A7pc_l | postcentral area BA 7, L | 0.832 ± 0.062 | 0.464 ± 0.047 | 4.69e-05 |
| A7pc_r | postcentral area BA 7, R | 0.719 ± 0.058 | 0.418 ± 0.047 | 3.68e-05 |
| A7ip_l | intraparietal area BA 7 (hIP3), L | 0.614 ± 0.055 | 0.303 ± 0.049 | 4.02e-05 |
| A40rd_l | rostro-dorsal area BA 40 (PFt), L | 0.815 ± 0.051 | 0.477 ± 0.042 | 5.07e-05 |
| A40rd_r | rostro-dorsal area BA 40 (PFt), R | 0.695 ± 0.048 | 0.420 ± 0.044 | 4.03e-05 |
| A40rv_l | rostro-ventral area BA 40 (PFop), L | 1.367 ± 0.058 | 0.992 ± 0.040 | 5.48e-05 |
| A40rv_r | rostro-ventral area BA 40 (PFop), R | 1.130 ± 0.042 | 0.893 ± 0.044 | 3.55e-05 |
| A7m_l | medial area BA 7 (PEp), L | 0.549 ± 0.070 | 0.274 ± 0.040 | 3.67e-05 |
| A7m_r | medial area BA 7 (PEp), R | 0.579 ± 0.075 | 0.309 ± 0.038 | 3.57e-05 |
| A5m_l | medial area BA 5 (PEm), L | 0.867 ± 0.061 | 0.513 ± 0.042 | 4.97e-05 |
| A5m_r | medial area BA 5 (PEm), R | 0.803 ± 0.055 | 0.516 ± 0.041 | 4.19e-05 |
| A1_2_3ulhf_l | area 1/2/3 (upper limb, head and face region), L | 0.719 ± 0.064 | 0.424 ± 0.043 | 3.94e-05 |
| A1_2_3ulhf_r | area 1/2/3 (upper limb, head and face region), R | 0.712 ± 0.064 | 0.428 ± 0.045 | 3.60e-05 |
| A2_l | area BA 2, L | 0.745 ± 0.057 | 0.382 ± 0.043 | 5.04e-05 |
| A2_r | area BA 2, R | 0.682 ± 0.062 | 0.392 ± 0.047 | 3.61e-05 |
| A1_2_3tru_l | area1/2/3 (trunk region), L | 0.820 ± 0.056 | 0.475 ± 0.044 | 4.79e-05 |
| A1_2_3tru_r | area1/2/3 (trunk region), R | 0.799 ± 0.067 | 0.510 ± 0.043 | 3.75e-05 |
| A23v_r | ventral area BA 23, R | 0.458 ± 0.078 | 0.183 ± 0.036 | 3.64e-05 |
| lsOccG_l | lateral superior occipital gyrus, L | 0.589 ± 0.061 | 0.342 ± 0.038 | 3.60e-05 |

* Comparing with the healthy group, all functional edges significantly increased after network based statistics (NBS) correction (significant p level = 1 x 10^-4^). SEM = standard error of mean. BA = Brodmann area.

# Supplementary Figures

# Supplemental figure. 1.

**
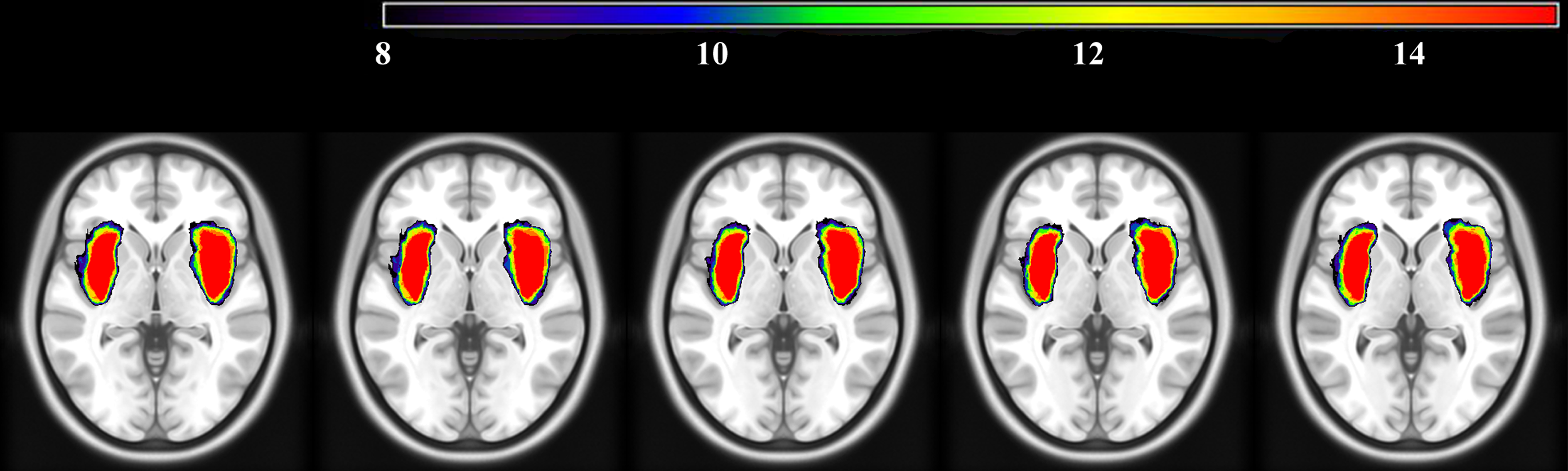
**

**Supplemental figure. 1.** Tumor location of the recruited patients. The color bar shows the number of tumor overlapping.

**Supplemental figure. 2.**

**
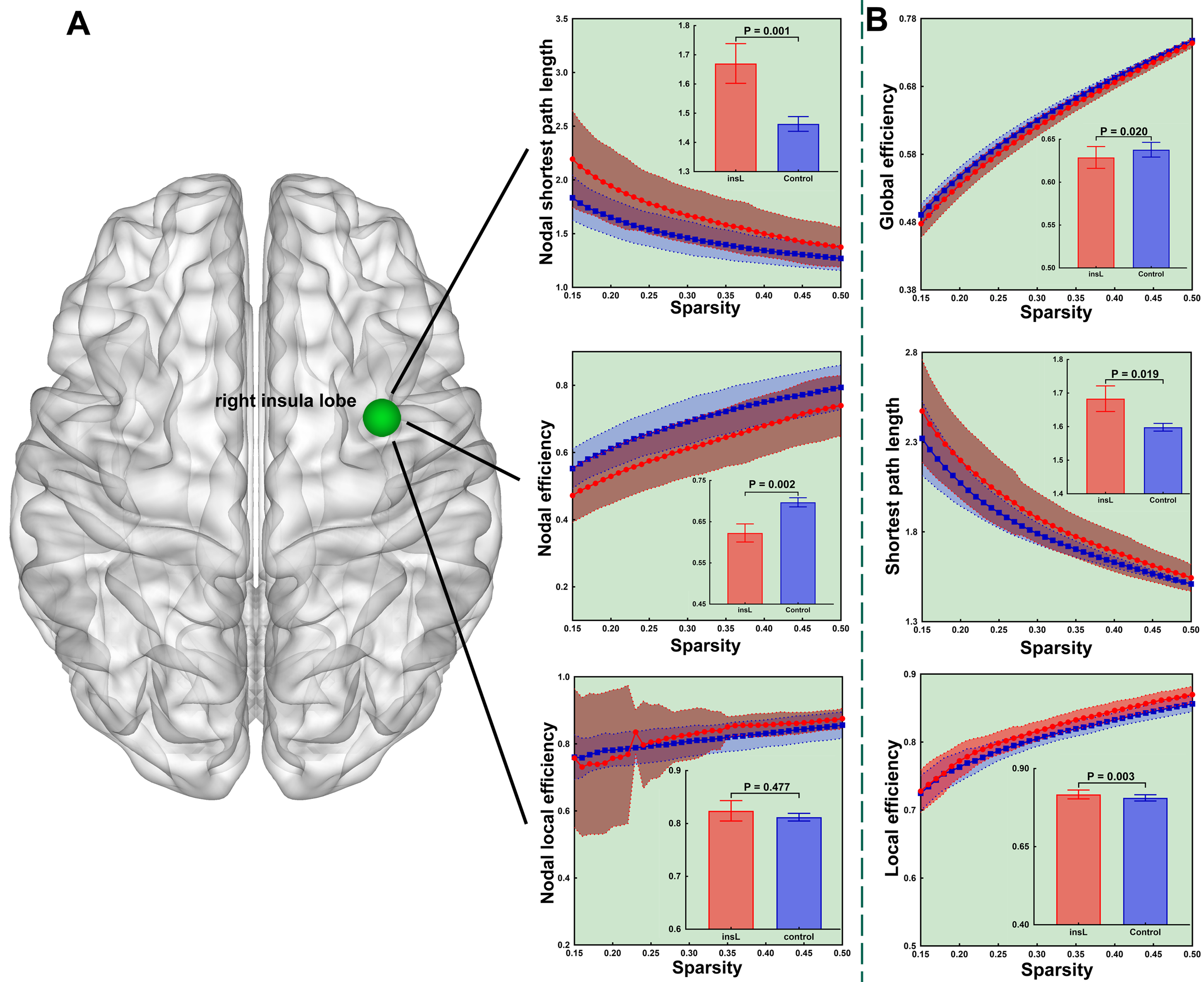
**

**Supplemental figure. 2.** In the healthy hemisphere template, compared to those of the healthy group, altered topological properties were observed in the insL group. A) Altered topological properties at the local level of insular lobe node. B) Altered topological properties at the global level. Light green node = right insular lobe.

**Supplemental figure. 3.**

**
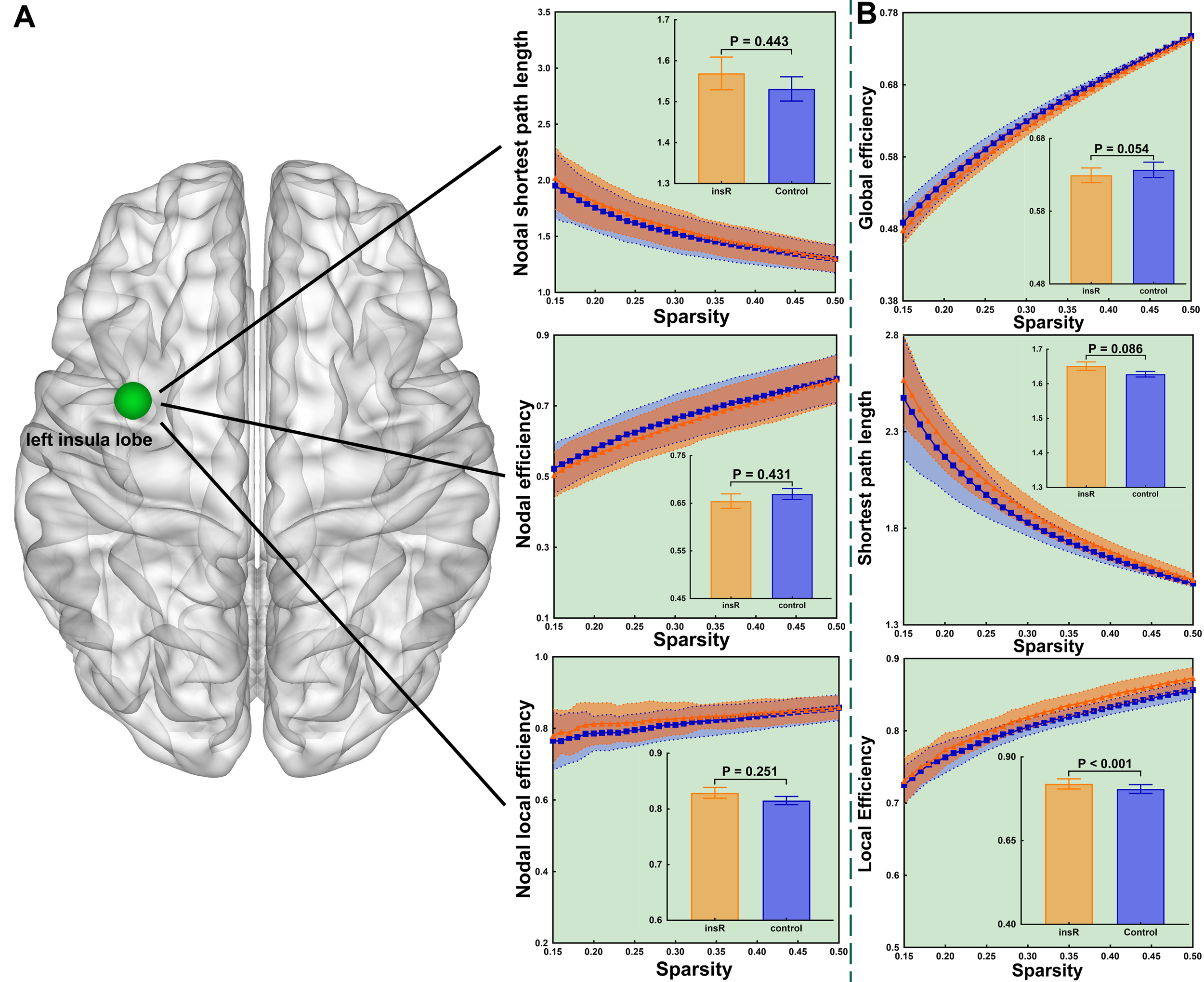
**

**Supplemental figure. 3.** In the healthy hemisphere template, compared to those of the healthy group, altered topological properties were observed in the insR group. A) Altered topological properties at the local level of the insular lobe node. B) Altered topological properties at the global level. Light green node = left insular lobe.
